# Validation and Optimization of Ultrasound-Assisted Dispersive Liquid-Liquid Microextraction as a Preparation Method for Detection of Methadone in Saliva with Gas Chromatography-Mass Spectrometry Technique

**DOI:** 10.34172/apb.2020.040

**Published:** 2020-02-18

**Authors:** Ahmad Shekari, Mehdi Forouzesh, Roohollah Valipour, Fardin Fallah, Pardis Shojaei

**Affiliations:** Legal Medicine Research Center, Legal Medicine Organization, Tehran, Iran.

**Keywords:** Dispersive liquid-liquid microextraction, Gas chromatography-mass spectrometry, Ultrasound, Methadone, Saliva, Validation

## Abstract

***Purpose:*** We investigated validation and optimization of ultrasound-assisted dispersive liquidliquid microextraction (UADLLME) as a preparation method for detection of methadone in saliva samples.

***Methods:*** We used blank and methadone-containing saliva samples and also standard methadone solution. Sodium hydroxide and chloroform were added to samples and they were held in ultrasonic bath. Then preparations were centrifuged and extracted analyte was analyzed by gas chromatography-mass spectrometry (GC-MS). Accuracy was measured by Intra and between-day mean relative errors (RE). Precision was assessed by coefficient of variation (CV). Recovery, specificity, linearity and limits of detection and quantification were also determined. Optimization was conducted for ultrasound duration, pH and extraction phase volume. Efficiency of dispersive liquid-liquid microextraction (DLLME) and UADLLME were compared.

***Results:*** Intra and between-day accuracies (2.3 -7.5%), recovery (89.4-115.5%) and precision (5.2-11.3%) were all acceptable. Calibration curve was linear in the concentration range of 150 ng/mL-10 µL/mL with R^2^ >0.9995 and equation of y=86.901x-5342.5. Limits of detection and quantification were 50 and 150 ng/mL, respectively. Specificity was measured by comparing retention times of saliva samples (containing methadone metabolites and other commonly used drugs) during UADLLME/GC-MS analysis and no interference was observed. Recovery of UADLLME was 1.4 of DLLME. Solvent and sample volumes required for UADLLME were 1/200 and 1/20 of DLLME. The greatest efficiency obtained at pH of 10, with ultrasound treatment duration of 5 minutes and extraction phase volume of 1000 µL.

***Conclusion:*** Study found that UADLLME/GC-MS is a valid and efficient method for detection of methadone in oral fluid.

## Introduction

Investigating biological samples for drugs has a fundamental role in forensic medicine and clinical toxicology.^1^ For efficient extraction of intended substances, biological samples need to be prepared before chemical analysis.^1,2^ Dispersive Liquid-liquid microextraction (DLLME) is a fast, easy, cost-effective and environmentally friendly preparation method that can be followed by Gas chromatography-mass spectrometry (GC-MS) analysis.^1,3^ Studies show that use of ultrasound waves during DLLME rises its efficiency and speed and reduces the required volume of toxic solvents.^4^

Rate of methadone consimption in Iran is high and rapid detection of this drug in biological samples is a requirement in forensic medicine and toxicology.^5^ In our forensic laboratories, DLLME/GC-MS is the routine method for methadone detection which is typically conducted on urine samples.^2^ Oral fluid (saliva) can be a good substitute for urine sample because of its easy collection, low probability of adultration and high accuracy for methadon detection.^5^

Limited resources in our forensic laboratories and increasing demand for methadone testing in Iran made us to seek for a more efficient, rapid and cost-effective technique of methadone analysis in biological samples as an alternative to current methods. Hence, we conducted qualitative validation of ultrasound assisted DLLME combined with GC-MS technique (UADLLME/GC-MS) for methadone detection in saliva samples and compared the efficiency of this method with conventional DLLME/GC-MS.

## Materials and Methods

### Chemicals and instrumentations

#### Chemicals

Standard methadone (as the main metabolite) with concentration of 100 µg/mL was bought from Sigma Aldrich, St. Louis, USA.

#### Instrumentations

GC-MS device: Agilnet (Model 7890, USA); Ultrasonic bath (305 Watts, 39 KHZ): Soltec, Italy

### Sample preparation by UADLLME

#### Sampling

After chewing gum, 4 mg of saliva was obtained from 50 drug abusers receiving methadone maintenance therapy at a private clinic. Fifty blank saliva samples were also obtained from staff of Legal Medicine Organization without any drug use during the month previous to sampling. Samples were collected in standard plastic containers and held in refrigerator without any preservative.

#### UADLLME method

At first step, 15 µL of sodium hydroxide (4 molar) was added to 1 mL of saliva samples to reach pH of 10 and these preparations were held at ultrasonic bath. Then, 1 cc chloroform was added to preparations and after 1-minute stirring, they were put at ultrasonic bath for 5 minutes. Third step was centrifugal of the preparations that led to separation of the aqueous and organic phases. Finally, aqueous phase was discarded and remained organic phase was slowly dried by nitrogen flow. Dried residues were dissolved in 100 µL of methanol and filtered. The filtered preparations were ready for GC-MS analysis.

### GC-MS analysis conditions

Temperature program: 60°C (1 minute), 210°C (5 minutes) @ 20°C/min, Injection volume: 0.5 cc, Injector temperature: 250°C, injection method: Splitless, transfer temperature: 280°C, Column: HP-5MS (30 m*0.25 mm*0.25 µm), Carrier gas: helium 99.999%, Gas flow speed: 1.5 mL/min, Detection method: Full scan for qualitative analysis of alkaline drugs and selected ion monitoring at m/z 72, 73, 91, 293 for methadone analysis.

### Optimization of UADLLME:

#### Ultrasound duration, pH and extraction phase volume

Saliva preparations containing 500 ng/mL methadone (500 µL of blank saliva sample was added to standard 1 µg/mL methadone solution) were treated with ultrasound for 1, 3, 5, 7 and 9 minutes and also at pH of 8, 9, 10 and 11. In addition, method was optimized for the volume of extraction phase in the range of 400-2000 µL. Test was repeated 3 times at each point and extraction efficiency was assessed by calculating the mean area under peak chromatograms.

### Validation of the UADLLME

Validity of the method was investigated by calculating accuracy, precision, recovery, linearity, specificity (selectivity), limit of detection (LOD) and limit of qualification (LOQ) (6).

#### Accuracy, precision and recovery

By spiking 3 concentrations of methadone (200, 800 and 7000 ng/mL) to blank saliva samples, we prepared three standard solutions and each was divided into 5 samples. Each of these 15 samples was analyzed 5 times a day and also during 5 consecutive days. We used following formulas for calculating main validation indicators:

Accuracy: Relative error (RE%): [(Measured concentration-real concentration/Real concentration)*100]. ^6^

Precision: Coefficient of variation (CV%): [(Standard deviation/Measuredconcentration)*100]. ^6^

Recovery: (Measured concentration / Added concentration)*100. ^6^

#### Calibration curve and linearity

Five methadone preparations with concentration range of 150 ng/mL-1000 µL/mL were analyzed by the proposed method and areas under the peak chromatograms were plotted against the real analyte concentrations to make the calibration curve. Linear regression analysis of the calibration curve was also conducted.

#### Specificity

Specificitywas determined by comparing retention times (RTs)^7^ of commonly used drugs and also main metabolites of methadone. Saliva samples positive for mentioned substances (confirmed by conventional DLLME/GC-MS) were selected and their RTs were measured by UADLLME/GC-MS and probable interferences in extraction were investigated.

#### LOD and LOQ

Blank samples show some concentration of analyte called analytical noise.^8^ LOD is the lowest analyte concentration which is distinguishable from analytical noise and LOQ is the lowest concentration at which analyte can be detected with reasonable precision and accuracy.^8^ For determination of analytical noise, three blank samples were analyzed by UADLLME/GC-MS and their mean concentrations (during the intended retention time) were regarded as the noise. Standard solution with lowest concentration was diluted consecutively and spiked to saliva samples and signal of each spiked sample was analyzed. The concentrations of solution with the signal-to-noise ratio of 3 and 10 were regarded as LOD and LOQ, respectively.^8^

### Comparing DLLME and UADLLME

Same sample with 500ng/mL methadone was analyzed by both DLLME/GC-MS and UADLLME/GC-MS methods and obtained peak chromatogram areas were compared.

## Results and Discussion

### Optimization

**Figure 1:**
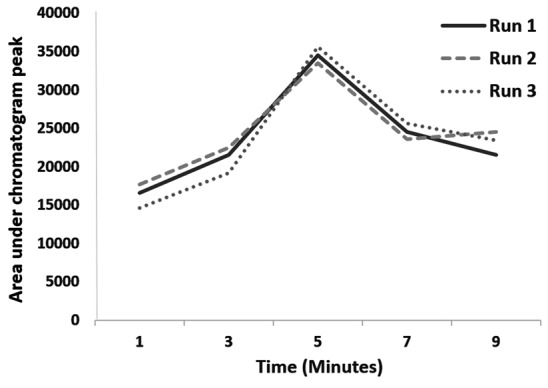
Optimization for ultrasound duration of ultrasound-assisted dispersive liquid-liquid microextraction/gas chromatography-mass spectrometry of methadone in saliva samples.

**Figure 2:**
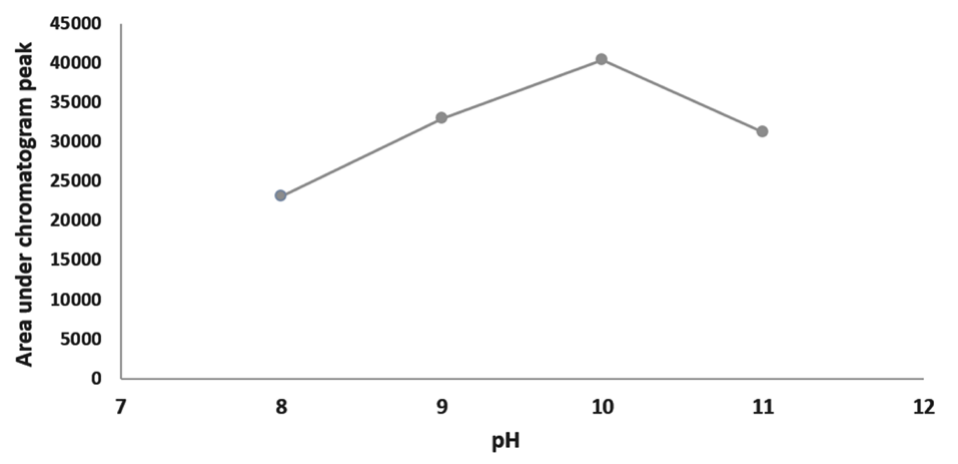
Optimization for pH of ultrasound-assisted dispersive liquid-liquid microextraction/gas chromatography-mass spectrometry of methadone in saliva samples.

**Figure 3:**
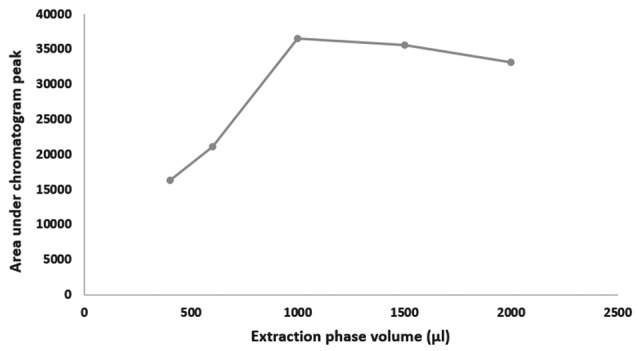
Optimization for extraction phase volume of ultrasound-assisted dispersive liquid-liquid microextraction/gas chromatography-mass spectrometry of methadone in saliva samples.

Effects of ultrasound duration, pH and extraction phase volume on the efficiency of extraction are presented in Figures 1-3. The best extraction efficiency was obtained with ultrasound treatment of 5 minutes. Lin et al, revealed that optimized ultrasonication time for UADLLME/GC-MS analysis of methadone in whole blood was 2 minutes.^1^ Other works also show that the most efficient ultrasound duration in UALLE is in the range of 1-30 minutes and longer duration may damage the analyte structure.^9^ As a rule, extraction efficiency of alkaline drugs is optimized at alkaline pH^3^ and we also observed that increasing alkaline pH from 8 to 10 led to increasing efficiency of extraction and the best result was obtained at pH of 10. Other studies on urine^3^ and whole blood^10^ also showed that pH of 10 is best for methadone extraction because at this pH the drug is in its molecular form.^10^ In the present work, the best efficacy was at extraction phase volume of 1000 µL.

### Validation

Accuracy, precision and recovery of the developed method are summarized in Table 1. Intraday accuracy in terms of RE% was in the range of 2.3-6%. Between-day accuracy was in the range of 2.8-7.5%. Precision (CV%) was also lower than 7.9% in several assays. Based on valid references,^8^ obtained values of accuracy and precision are acceptable. Recovery was in the range of 89.4-115.5% which is also good according to guidelines.^11^ Lin et al,^1^ found similar results for precision, accuracy and recovery in the analysis of whole blood for methadone by UALLME/GC-MS.

**Table 1:** Accuracy, precision and recovery of ultrasound-assisted dispersive liquid-liquid microextraction/gas chromatography-mass spectrometry of methadone in saliva samples

**Assays**	**Added methadone (ng/mL)**	**Average measured methadone (ng/mL)**	**Range of measured methadone (ng/mL)**	**SD of measured methadone (ng/mL)**	**Accuracy(RE %)**	**Precision (CV%)**	**Recovery (%)**	**Recovery range (%)**
Intraday	200	212	193-231	16.7	6	7.9	106	96.5-115.5
	800	842	790-910	45	5.2	5.3	105	98.7-113.7
	7000	6833	6700-6950	121	2.3	1.8	97.6	95.7-99.3
Between-day	200	215	210-225	6.5	7.5	3	107.5	105-112.5
	800	754	715-786	25.7	5.7	3.4	94.2	89.4-98.2
	7000	6811	6698-6952	96.7	2.8	1.4	97.3	95.7-99.3

SD, standard deviation; RE, relative error; CV, coefficient of variation.

Calibration curve was linear (R^2^=0.9995) in the wide concentration range of 150-10000 ng/mL of methadone with the following equation: y=86.901x-5342.5.

**Figure 4:**
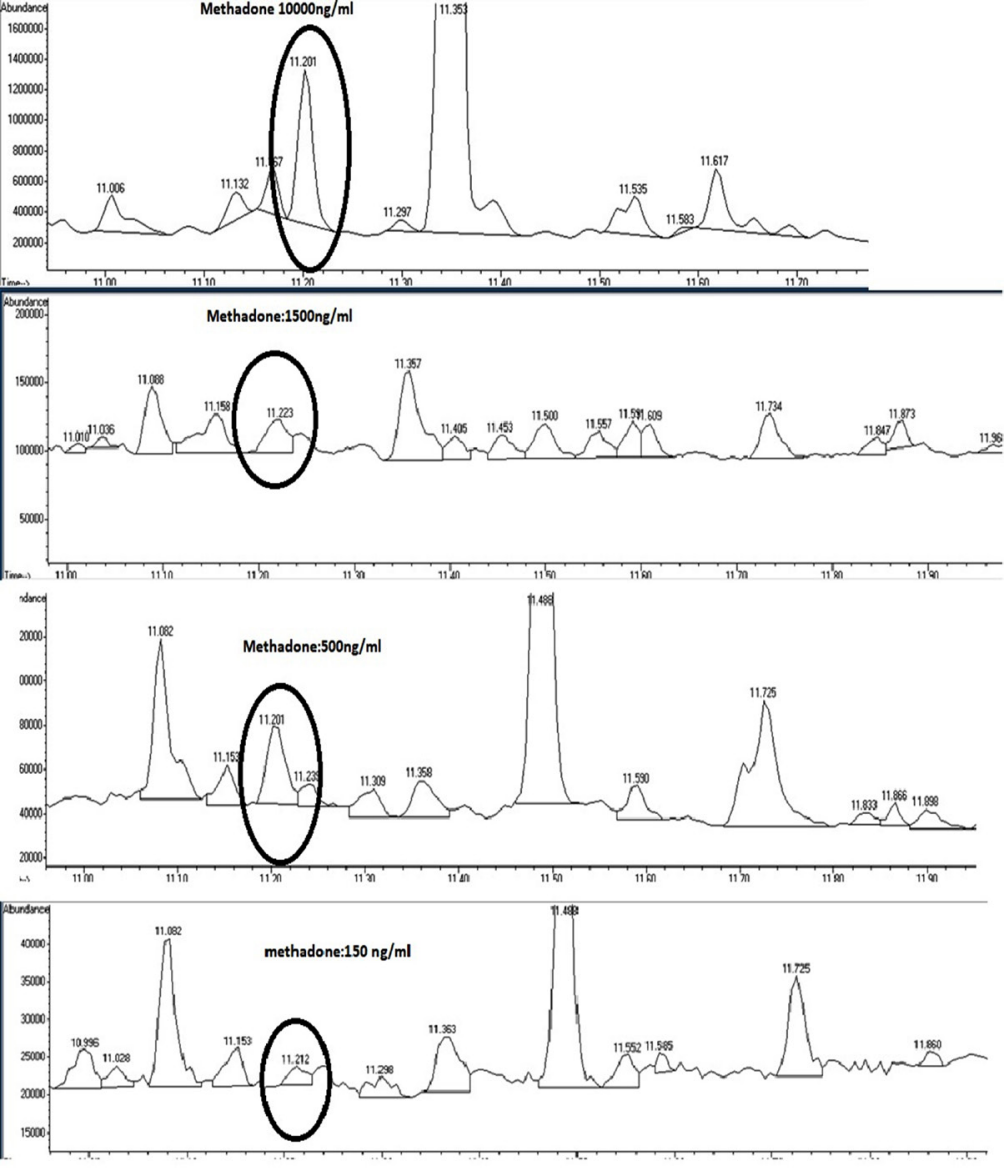
Chromatogram of various methadone concentrations (used for calibration curve) during Ultrasound-assisted dispersive liquid-liquid microextraction/gas chromatography-mass spectrometry of methadone in saliva samples.

Chromatograms of concentrations used for linear regression analysis are shown in Figure 4.

Table 2 compares RTs of several commonly used substances and methadone metabolites. No interferences were observed and method was specific. LOD and LOQ of the UADLLME/GC-MS analysis for methadone were 50 and 150 ng/mL, respectively.

**Table 2:** Retention times of saliva samples containing various substances during Ultrasound-assisted dispersive liquid-liquid microextraction/gas chromatography-mass spectrometry

**Drugs**	**Number of Samples**	**Mean Retention time (min)**
None (Blank)	7	--
Methadone	15	11.22
EDDP	13	10.72
EMDP	11	10.11
Tramadol	10	10.26
Caffeine	3	9.82
Nicotine	2	6.63

### Comparing LLE and UALLE

Peak chromatogram area for analysis of a similar sample with 500ng/mL of methadone was 375567 for DLLME compared with 523354 for UADLLME. Solvent and sample required for UALLE were 1/200 and 1/20 of them in conventional DLLME. Other relevant studies have also demonstrated such advantages for the use of ultrasound during extraction.^1,12^

There were several limitations to this study. We tried to improve conventional methods for methadone detection in forensic laboratories with regard to cost-effectiveness; thus we used chloroform which is the main solvent in our laboratories but other solvents should also be tested in UADLLME. To generalize the proposed method, validation of other alkaline substances should be investigated which was not possible due to our financial limits. Several issues such as temperature, salts, added ions and buffers affect the efficiency of the method that were not investigated in the present work and should be regarded in future studies.

## Conclusion

Findings of this study endorse the validity and efficiency of UADLLME/GC-MS analysis of methadone in oral fluid. This method needs lower solvent and provides greater recovery, compared to DLLME/GC-MS and can replace the conventional analysis of methadone in our forensic laboratories.

## Ethical Issues

Samples were analyzed without any identity of donors.

## Conflict of Interest

Authors declare no conflict of interest in this study.
